# Diet in pregnancy—more than food

**DOI:** 10.1007/s00431-017-3026-5

**Published:** 2017-11-03

**Authors:** H. Danielewicz, G. Myszczyszyn, A. Dębińska, A. Myszkal, A. Boznański, L. Hirnle

**Affiliations:** 1 1st Department of Pediatrics, Allergy and Cardiology, Wroclaw Medical University, Chalubinskiego 2a 50-368, Wroclaw, Poland; 2 1st Department of Obstetrics and Gynecology, Wroclaw Medical University, Chalubinskiego 3 50-368, Wroclaw, Poland

**Keywords:** Pregnancy, Maternal diet, Developmental programming

## Abstract

High food quality, together with adequate macro- and micronutrient intake in pregnancy, is crucial for the health status of the mother and child. Recent findings suggest that it could also be beneficial or harmful in the context of the well-being of the whole future population. According to the developmental origins of health and disease hypothesis, most conditions that occur in adulthood originate in foetal life. Moreover, some epigenetic events, modified inter alia by diet, impact more than one generation. Still, the recommendations in most countries are neither popularised nor very detailed. While it seems to be important to direct diet trends towards a healthier lifestyle, the methods of preventing specific disorders like diabetes or asthma are not yet established and require further investigation.

*Conclusion*: In this review, we will summarise the recommendations for diet composition in pregnancy, focusing on both diet quality and quantity.

**Table:** 

**What is Known**
• *High food quality, together with adequate macro- and micronutrient intake in pregnancy, is crucial for the health status of the mother and child.*
**What is New**
• *Recent findings suggest that the diet could be beneficial or harmful in the context of the well-being of the whole future population. Most conditions that occur in adulthood originate in foetal life.*
• *Moreover, some epigenetic events, modified by diet impact more than one generation.*

## Introduction

The substantial increase in the prevalence of common diseases like asthma, atopy, obesity, hypertension and diabetes observed over the past decades has directed attention to specific changes in the environment as a possible cause of such an unfavourable switch. Among environmental factors, the diet is a crucial influencer of population health. According to the developmental origins of health and disease hypothesis, most conditions that occur in adulthood originate in foetal life. Pregnancy is a specifically “hot period” for the programming of future condition. The relevance of the maternal diet to serious pregnancy outcomes such as preeclampsia, hypertension, preterm birth and fertility has also been revealed [23, 26].

In this narrative review, we will summarise the recommendations for diet composition in pregnancy and existing developmental theories, focusing on both diet quality and quantity. The aim of this review is to give the interpretative synthesis of the current knowledge and highlight the developmental aspect of maternal diet.

The literature search was provided via PubMed database with the following search terms: “diet in pregnancy recommendation”, “specific micro- or macronutrient and pregnancy”, “specific pregnancy outcome”, “diet in pregnancy and atopy/asthma”, “developmental origin of disease” focusing on both more recent reviews limited to specific aspects of diet and original papers.

### Composition of the maternal diet—quality

Specific recommendations exist for different types of nutrients in pregnancy. They differ in some points according to both the eating tradition and nutrition status of the population. WHO antenatal standards paper provides 39 recommendations related to 5 types of interventions. The healthy eating and physically active style of life is promoted to prevent excessive gestational weight gain (GWG). In the undernourished population, balanced energy and protein intake are recommended to prevent LBW, SGA, and stillbirths. Doses of iron and folate supplementation are given with possible daily or intermittent routine. Supplementation of vitamin A is suggested to be restricted only to areas where vitamin A deficiency is a substantial public health problem. Recommendation of calcium supplementation is limited to population with low-calcium intake. Vitamin B6, zinc, multi-nutrient supplements and vitamin D supplementation are not advocated as routine procedure. Avoiding of caffeine is suggested for women with high consumption [40]. Canadian consensus highlights the need of the uptake of nutrient-dense and energy-appropriate food with moderate increase of energy intake during pregnancy. Particular concern is given to GWG, adequate folate, iron, choline, omega-3 fatty acid and iodine input, as well as avoiding or limiting specific food which contains bacteria or methyl mercury and alcohol [25]. German National Consensus is quite detailed in different aspects of diet in pregnancy. In the first paragraph, the difference between slightly increase of energy needs in comparison to a much greater increase of vitamin and minerals is highlighted. According to these requirements, nutrient-dense food eating, regular meals and regular exercises together with moderate GWG are recommended. The specific concerns exist for obese pregnant women for whom the standards of care and weight lose still are not well established, vegetarian nutrition with possible supplementation of iron and DHA and vegan where specific medical counselling is required due to diet deficiency of many nutrients [16]. Italian Consensus differs a little in the points according to energy input and protein intake during pregnancy, where specific amounts are recommended in the particular periods. The emphasis is put on the protein and fat composition, iron supplementation, as well as iodine and calcium adequate provision [17]. Standards of nutrition for Polish population, reflecting WHO and EFSA recommendations, contain tables for different groups according to age, sex and pregnancy status for both micro- and macronutrients together with energy requirements and expenditure [7, 14]. Similar tables are published by Institute of Medicine [13, 31]. Further in the text, the nutrient requirements during pregnancy are described in details and summarised in Table 1. Apart from the recommendations, there is substantial body of reviews concerning specific aspects of maternal nutrition. In the last 2 years, we identified important papers in the subject relating to diet and fertility, interventions for diabetic or obese pregnant women, metabolic consequences of excessive GWG, the impact of the diet rich in polyphenols, the use of probiotics and prebiotics, the maternal microbiome and the development of neonatal immune system, the benefits of Mediterranean diet and the epigenetic programming.

**Table 1:** Micro- and macronutrients intake during pregnancy—summary of the recommendations

Energy	No additional input I trimester 340 kcal/day II trimester 452 kcal/day III trimester [31] 69 kcal/day I trimester 266–360 kcal/day II trimester 437–496 kcal/day III trimester [17] 10% increase in late pregnancy—260 kcal/day [16]
GWG	• BMI < 18.5 kg/m^2^ GWG 12.5–18 kg • BMI 18.5–24.9 kg/m^2^ GWG 11.5–16 kg • BMI 25–29.9 kg/m^2^ GWG 7–11.5 kg • BMI > 30 kg/m^2^ GWG 5–9 kg [25, 40]
Protein	10–35% of energy, 71 g/day [13] Additional 1 g/day I trimester 8 g/day II trimester 26 g/day III trimester [17] RDA 1.1 g/kg/day [25] RDA 1.2 g/kg/day [14]
Carbohydrates	45–65% of energy, 175 g/day
Fat	20–35% of energy [13] Additional 8–14 g/d II trimester 11–18 g/day III trimester [14]
n-6	13 g/day, 5–10% [13]
n-3	1.4 g/day, 0.6–1.2% [13] EPA 250 mg/day DHA 100–200 mg/day [14, 16] DHA 600–1000 mg in risk groups [7]
Fibre	28 g/day [13, 31]
Iron	Supplementation 30–60 mg/day [40] RDA 27 mg/day [14, 31]
Iodine	RDA 220 mcg/day [14, 31] Supplementation 100–150 mcg/day [16] Supplementation 200 mcg/day [7] None additional supplementation [40]
Folate	RDA 600 mcg/day [31] Supplementation 0.4 mg/day [7, 16, 40]
Calcium	RDA 1.0–1.3 g/day [31] Supplementation 1.5–2 g/day in risk population (low calcium intake) [40]
Vitamin D	RDA 5 mcg (200 IU)/day [31] RDA 15 mcg (600 IU)/day [17] At least 600 IU/day RDA, 1500–2000 IU/day to maintain the level above 30 ng/ml [11] None additional supplementation in general [40] Additional supplementation in risk groups 2000 IU/day [7]

### Macronutrients

#### Protein

Both the quantity and the composition of protein are important in the context of diet quality. In a rat model, protein deficiency in pregnancy results in decreased birth weight, decreased heart weight, increased heart rate and increased systolic blood pressure [2]. In general, animal protein is of higher quality than vegetable protein, suggesting that meat should be the main source of protein in pregnancy, but mixing different types of vegetables increases the quality of plant protein substantially.

Nevertheless, it should also be considered that specific types of plant diets, such as vegetarian and vegan diets, are associated with microelement and mineral deficiencies and unfavourable pregnancy outcomes. In this context, a vegetarian diet can result in vitamin B12 and iron deficiency, as well as low birth weight, whereas a vegan diet can lead to inadequate intake of DHA, zinc and iron, as well as an increased risk of preeclampsia and inadequate brain development. However, still a well-balanced ovo-lacto vegetarian diet usually enables good nutrient status in pregnancy, when supplemented with vitamin D, folic acid, iodine, iron, vitamin B12 and zinc, and, in cases of a fish-free diet, with DHA [19].

In contrast, consumption of red meat, which was recently revealed to be associated with cancer risk, raises some concerns over pregnancy and protein requirements, but till now, there are no any evidences that this diet can negatively impact child’s health [24].

#### Fat

Fat in the diet of pregnant woman is important mainly in context of fatty acid composition, mainly that of DHA and eicosapentaenoic acid (EPA). Omega-3 fatty acids are beneficial for brain development and proper functioning of the retina. In many studies, maternal serum DHA concentration has been associated with neuronal development and plasticity, receptor-mediated signalling, membrane fluidity and the formation of second messengers. This type of fatty acid also impacts modulation of inflammation by affecting Toll-like receptors (TLRs), related to adequate response to bacteria and other microorganisms. DHA also plays a role as a precursor of the anti-inflammatory lipid mediator RvD, which prevents the formation of proinflammatory arachidonic acid products, thus indicating the anti-inflammatory function of these molecules [33].

#### Carbohydrates

Carbohydrates are an essential component of a healthy diet. However, increased caloric intake associated with increased fat and carbohydrate consumption with adequate protein has been associated with neonatal adiposity, which is obviously unfavourable [28]. Additionally, a preconception diet rich in saturated fat, carbohydrates and take-away food has been associated with poor asthma control during pregnancy, thus affecting child well-being [8]. Moreover, changing the maternal eating pattern by decreasing carbohydrate load and increasing physical activity could impact the inflammation status associated with obesity in pregnant women [32]. Similarly, modifying the protein/carbohydrate ratio can decrease the expected GWG [18].

#### Fibre

The main role of fibre is to modulate gut microbiome. A high-fibre diet has been shown to prevent asthma by epigenetic switch and by impacting the gut microbiota. In a mouse model, a diet differing only in fibre amount with the same fat, protein, carbohydrates, energy and weight gain impacts the development of allergic airway disease (AAD; a model of human asthma). The mechanism is believed to be related to the specific microbiota and level of short-chain fatty acids (SCFAs) in faeces and serum. Specifically, SCFAs (acetate, propionate and butyrate) regulate acetylation of Foxp3 and Treg development and thus have an anti-inflammatory effect but also affect epithelial integrity. The SCFA propionate also impacts dendritic cell (DC) biology and the ability to promote the T helper 2 (Th2) response and regulates NPPA gene expression in the lungs. All these phenomena happen only in foetal life, possibly in the early stages of development, but even later in pregnancy has some impact, where the high-fibre diet has been shown to correlate with fewer GP visits in the first year of life due to cough and wheezing [37].

### Micronutrients

#### Iron

Iron is one of the most important micronutrients. The usual absorption from plants is low and could be further decreased by phytates and polyphenols, which are present in some plant-based products. The absorption of haem iron from meat is much higher.

Inadequate iron intake during pregnancy is associated with cardiovascular risk to the offspring in adulthood. In animal models, maternal iron deficiency has been associated with obesity, hypertension and adverse cardiovascular outcomes [1, 29].

#### Iodine

Iodine is another very important micronutrient. Iodine deficiency has been revealed to be associated with postpartum hyperthyroidism, perinatal mortality and neonatal hypothyroidism. Inadequate iodine intake during pregnancy causes an increased risk of spontaneous abortion, higher mortality, birth defects, neurological disorders and brain damage [10]. Fish and shellfish, fruits, vegetables, milk, eggs and meat are the main source of iodine from the usual diet.

#### Calcium and vitamin D

The main source of calcium is milk and milk products (50%), cereals (11%) and vegetables (11%). It is crucial for bone metabolism but also related to birth weight, risk of preterm labour and appropriate blood pressure [12].

Early studies concerning vitamin D in pregnancy showed an association with preeclampsia and caesarean section but also glucose tolerance, abnormal foetal grown pattern, preterm birth and reproductive failure. In the first weeks of pregnancy, the level of the vitamin D metabolite 1,25(OH)D3 increases 2–3-fold, regardless of the level of intake, but the significance of this phenomenon is unknown. This mechanism could possibly maintain the required level during pregnancy if preconception stores were normal. Below adequate levels of 25(OH)D3 (< 20 ng/ml) are related to adverse outcomes later in life, such as asthma, multiple sclerosis, neurological disorders and autoimmune conditions.

The main dietary sources of vitamin D are cod liver oil and fish. Smaller amounts are present in eggs, butter and cheese; however, the most important contributor to the general level is skin production upon exposure to UV radiation and additional supplementation [21].

#### Folates

Folates are extremely important for the prevention of neural tube defects. The RDA increases by up to 50% in pregnancy, and the recommended supplementation dose is 400–800 μg from 2 months prior to conception onward, which is essential in the first trimester and could be continued after the 12th week of pregnancy [39].

#### BPA

Environmental exposure to harmful substances in pregnancy, especially those present in the diet, raises concerns. BPA is used for different types of food packaging and as food additives and has now become the focus of interest. Exposure to this substance has been associated with adiposity, energy balance [38] and neurogenesis [22] and thus can be related to obesity and neurological disorders such as ADHD, anxiety, depression and sexual dimorphic behaviours.

### Composition of the maternal diet—quantity

#### Gestational weight gain

According to US epidemiological data, 69% of the population is overweight and 35% is obese. This change in prevalence is related to changes in lifestyle, but some prenatal events are also important. Gestational weight gain GWG has been shown to be a predictor of pregnancy complications and future health problems in the child [15, 30]. GWG is strongly associated with birth weight and values exceeding 4000 g are associated with a 2-fold greater risk of obesity later in life. Excess intake of calories during pregnancy has been associated with miscarriage, diabetes and preeclampsia in mothers and obesity and type 2 diabetes in children. This diabetic effect seems to be transgenerational. The mechanism is possibly related to placental gene-expression changes [35].

Surprisingly, opposite effect was described by Barker and colleagues who observed that nutritional insufficiency in the foetal period reflected by LBW or SGA is also related to glucose intolerance, diabetes, hypertension and coronary disease later in life [3]. These observations are the basis of the so-called thirsty phenotype hypothesis, which reflects the changes in the metabolism as it increases efficiency. The values for appropriate GWG are given in Table 1.

### Metabolic programming, cardiovascular risk and cortisol metabolism

A substantial number of studies have proven the causative relationship between birth weight, weight catch-up and cardio-metabolic risk reflected by alternation in liver and pancreatic functions [36]. “Fatty liver” in normal-weight offspring could be both a result of a high-fat maternal diet or protein restriction. Later in life, both poor weight gain and accelerated weight gain in the first months of infancy increase the risk of non-alcoholic fatty liver disease (NAFLD) [27]. What is more, maternal protein restriction in animal models modifies the offspring’s islet cell ontogeny and the number of beta cells [5, 34]. In humans, LBW is associated with pancreatic beta cell hyperplasia, and SGA causes a reduction of beta cell number. Consequently, SGA or LBW results in insulin resistance or diabetes in adulthood. Rapid weight catch-up during early infancy in children with LBW increases the risk of unfavourable metabolic events [4].

On the other hand, altering the foetal neuroendocrine environment specifically by impacting on the ACTH/cortisol level affects brain development. The foetus could be protected against increased levels of maternal cortisol by the placental enzyme 11β-HSD. This barrier, however, is disrupted by obstetric complications like preeclampsia and preterm birth as well as IUGR (intrauterine growth retardation), medication and diet. In an animal model, a maternal high-fat diet could increase anxiety in offspring by the interplay with serotonin, dopamine and HPA (hypothalamic-pituitary-adrenal) axis. In this model, also maternal anxiety, reflected by increased levels of cortisol, has been shown to cause impaired cognition, deficits in learning and memory, sex-atypical behaviours, heightened emotionality and general anxiety. It also impacts reactivity to stress and sensitivity to nicotine and other addictive substances. Rats prenatally stressed react with a faster, stronger and prolonged cortisol response later in life. In humans, a relationship between maternal stress in the third trimester and lower scores in attention and reactivity in newborns has been shown in some of them, as has a relation to Bayley Scales of Infant Development (BSID) and mental/motor development at 8 months. Data from epidemiological studies suggest links between maternal obesity and metabolic complications with neurological disorders like ADHD, ASD, schizophrenia, anxiety and depression [6, 20].

### Atopy and asthma programming

Perhaps not surprisingly, because food allergy is usually the first manifestation of atopy in life, atopy and asthma are conditions associated strongly with the maternal diet. Different diets have been studied in relation to this conditions risk. A diet focused on avoiding the main allergens was shown not to be related to atopic outcome in offspring. A holistic diet rich in a variety of foods is believed to be beneficial. Specifically, a diet rich in fish oil and PUFAs, probiotics, antioxidants and vitamins has been shown to be protective. Folate, a known methyl donor impacting methylation status, at specific doses has the reverse effect, due to the epigenetic mechanism. Specifically, high doses of folic acid (≥ 5 mg/day) in late pregnancy are an established risk factor for allergy. In contrast, nicotinamide, another methyl donor, decreases the risk of eczema at 12 months. Its main sources are vitamin B3 and tryptophan [9].

## Conclusion

Here, we tried to answer questions concerning pregnancy: What to eat? How much eat? Why is it important? Recommendations proposed by different authorities are based on the solid knowledge. However, there are some differences—population specific, they depend on the eating customs and tradition, and interventions which have been already introduced for the whole population. Some concerns exist for adequate folate supplementation, appropriate dose of DHA and iodine. Still, it seems to be difficult for ordinary pregnant woman to design proper diet. Novel electronic applications could be helpful; however, the algorithm should be approved by local health authorities. Hopefully healthy eating becomes trendy nowadays, which is the promise of good health for future population.

## Abbreviations

AAD: Allergic airway disease
ADHD: Attention-deficit hyperactivity disorder
ALA: Alpha linolenic acid
BMI: Body mass index
BPA: Bisphenol A
DC: Dendritic cell
DHA: Docosahexaenoic acid
EPA: Eicosapentaenoic acid
GPR20: G protein-coupled receptor 20
GWG: Gestational weight gain
HELP: Haemolysis, elevated liver enzymes and low platelet count
IL-1β: Interleukin-1beta
IL-6: Interleukin-6
IL-8: Interleukin-8
IOM: Institute of Medicine
IUGR: Intrauterine growth retardation
LBW: Low birth weight
LPS: Lipopolysaccharide
NF-κB: Nucleic factor kappa B
NPPA: Natriuretic peptide A
RDA: Recommended dietary allowances
RvD: Resolvin D
SCFA: Short-chain fatty acid
SGA: Small for gestational age
TLR: Toll-like receptor
TNFα: Tumour necrosis factor alpha
